# Non-Fickian Moisture Transport in Vegetable-Fiber-Reinforced Polymer Composites Using a Langmuir-Type Model

**DOI:** 10.3390/polym12112503

**Published:** 2020-10-27

**Authors:** Rafaela Q. C. Melo, Marcus V. Lia Fook, Antonio G. B. de Lima

**Affiliations:** 1 Department of Materials Engineering, Federal University of Campina Grande (UFCG), Campina Grande 58428-830, Paraiba, Brazil; marcus.fook@ufcg.edu.br; 2 Department of Mechanical Engineering, Federal University of Campina Grande (UFCG), Campina Grande 58428-830, Paraiba, Brazil; antonio.gilson@ufcg.edu.br

**Keywords:** water absorption, composites, vegetable fibers, finite volume, numerical simulation

## Abstract

The purpose of this article was to theoretically study the non-Fickian moisture absorption process in vegetable-fiber-reinforced polymer composites using a Langmuir-type model. Here, the focus was on evaluating the effect of the water layer thickness that surrounds the composite during the water migration process. The solutions of the governing equations were obtained using the finite volume method, considering constant thermophysical properties and non-deformable material. The results for the local and average moisture content and concentration, gradient values, and the transient rates of the free and bound (water) molecules in the process were presented and analyzed. It was observed that the water layer thickness strongly influenced the water absorption kinetics, the moisture content gradient values, and the equilibrium moisture content inside the material. It is envisaged that this new approach will contribute to better interpretation of experimental data and a better understanding of the physical phenomenon of water absorption, which directly affects the properties of composite materials.

## 1. Introduction

In recent years, growing environmental concerns have encouraged several industrial sectors to adapt their technologies and products to meet sustainable demands. Thus, the concept of “green materials”, or “eco-friendly materials”, has been standing out in the scientific and industrial fields [1]. In this sense, the use of vegetable fibers as reinforcement in polymeric matrix composites instead of conventional synthetic fibers presents numerous advantages, which may result in products with acceptable performance and costs and the potential for different applications [2,3,4].

However, the chemical structures of vegetable fibers makes the composites produced from them more sensitive to the influence of external environmental factors, such as temperature and moisture. Due to their polar and hydrophilic nature, when exposed to moisture, these materials will inevitably absorb water, causing swelling in the fibers and changes in their properties, decrease their durability during operation [5]. In this way, in-depth studies related to these phenomena are essential in order to clarify the consequences of moisture exposure on the mechanical, physical, and chemical properties of these composites and to find alternative solutions this important problem.

The diffusion of water molecules inside a composite material occurs by imperfections in its matrix, by capillarity along the interface, and by regions lacking polymeric resin, which proceeds until reaching the vegetable fibers. This phenomenon results in an increase in the free volume of the structure, which is responsible for changes in the properties of these materials. In addition, water absorption promotes fiber expansion and volume variations due to changes in moisture content, causing microcracks in the matrix. Thus, the prediction of the moisture content over time is particularly important, since from the obtained results it is possible to predict which areas are more susceptible to cracks and deformations, which directly affect the quality of the product, especially under moist environments applications [6,7].

To properly explain the macroscopic behavior of water molecules during the absorption process, it is necessary to have a set of experiments that faithfully reproduce the work conditions. However, each experiment usually takes several weeks, which is a limitation of the technique. Further, experimental approaches depend on predefined conditions that may inappropriately represent the real environment, affecting the achievement of reliable results and their interpretation. Alternatively, the use of appropriate and validated mathematical models allows the prediction of the material behavior in the long term quickly and economically, eliminating the need to condition the composites in humid environments. Therefore, several authors have conducted studies aiming to predict the behavior of these materials over long periods and under different conditions of moisture exposure [8,9,10,11].

The diffusion laws often explain the water-absorbing behavior of composite materials. Fick’s diffusion model is the most used in predicting moisture transport in composite materials [10,12,13,14,15,16,17], mainly for the initial moisture uptake region. The preference for this model is associated with the ease of fitting the governing equation to the experimental sorption data. However, for long periods, the moisture absorption in the material may not occur only by diffusion [18,19], whereby different processes such as chemical reactions can be activated, directly affecting the humidification kinetics and inducing a deviation in the behavior of the water absorption characteristic curve. In such cases, it is said that the humidification process is of the non-Fickian type, whereby it becomes necessary to use appropriate models that adequately capture these effects.

In this context, the Langmuir-type model [20] considers the fact that the absorption process occurs in two states. In the first state the water molecules are free to diffuse into the material, while in the second state these molecules become chemically bonded to the polymer chains. This process can be irreversible and means the non-Fickian behavior needs to be observed. The main advantage presented by Langmuir’s model as compared with Fick’s model is the fact that it considers the interaction between bound and free water molecules within the composite, being widely used to explain anomalous diffusion processes, and thus allowing for more consistent physical interpretations with real phenomena, since Fick’s model does not predict this deviation.

Based on the information cited before, some researchers have used the Langmuir-type model to describe the water absorption kinetics in vegetable-fiber-reinforced polymer composites. Santos et al. [21] presented a theoretical study of the anomalous behavior of moisture diffusion in composite materials reinforced with caroá fibers using Langmuir’s model. The predicted results of the average moisture content compared to experimental data showed that the model was effective in describing the phenomenon, allowing a better understanding of the moisture migration mechanisms. Chilali et al. [22] investigated the behavior of water diffusion in polymeric composites of thermoplastic and thermosetting matrices reinforced with flax fibers. The aim was to evaluate the effects of the fiber content, thickness, and orientation on the process behavior. Gravimetric tests were performed in pure water and salt water and the diffusion parameters were obtained using both Fick and Langmuir’s models. The authors concluded that both the models correctly described the transient evolution of water uptake. Meloetal [23] presented a review of polymeric composites reinforced with vegetable fibers and discussed the problem of moisture absorption in these materials. The authors reported analytical and numerical solutions for Langmuir’s modeling using a one-dimensional approach. The study was applied to caroá fibers and the authors obtained a good agreement between the theoretical and experimental data for the average moisture content throughout the process. Britoetal [24] developed a three-dimensional and transient mathematical model using Langmuir’s model and its numerical solution based on the finite volume method. This was used to predict the water absorption in polymeric composites reinforced with vegetable fibers. The obtained results were validated with experimental data for sisal fibers as reinforcement. The results for the concentrations of free and bound water molecules and the average and local moisture contents at different process times were presented, allowing the authors to describe in more detail the characteristics inherent to the absorption process. However, despite the importance of more precisely describing the water sorption phenomena inside the composite, which has not yet been explained by Fick’s model, there are still few studies focused on the mass transport mechanisms using Langmuir’s model, especially those that present new approaches to assess the influence of different test conditions on the absorption process.

An important component of the water sorption experiments in vegetable-fiber-reinforced polymer composites the sample selection. When placed in the water bath, if a sample has a higher density it will not float, rather it will sink to the bottom of the container, causing differences in the water absorption rate between the top and bottom sides of the sample. However, despite this phenomenon being observed in practical applications, when predicting this phenomenon, researchers must consider that the boundary conditions on the material surfaces are the same, contrary to the real process conditions.

In this sense, supplementing the research already carried out, this work aims to evaluate the effect of the water layer thickness that surrounds the composite during the water migration process using the Langmuir-type model. The one-dimensional solution for the governing equations was obtained numerically using the finite-volume method, considering the constant thermophysical properties and non-deformable material. These results were validated using the analytical solution published previously proposed by Santosetal [21], as applied to vegetable-fiber-reinforced polymer composites.

Thus, the innovative aspect of the research is the precise explanation of the distribution of the water inside the composite during the absorption and the velocity with which this phenomenon occurs. This information will assist engineers, researchers, and industries in making decisions related to the use of these materials in humid environments and in adequately understanding the absorption phenomenon.

## 2. Methodology Modelling

### 2.1. The Physical Problem and Geometry

**Figure 1:**
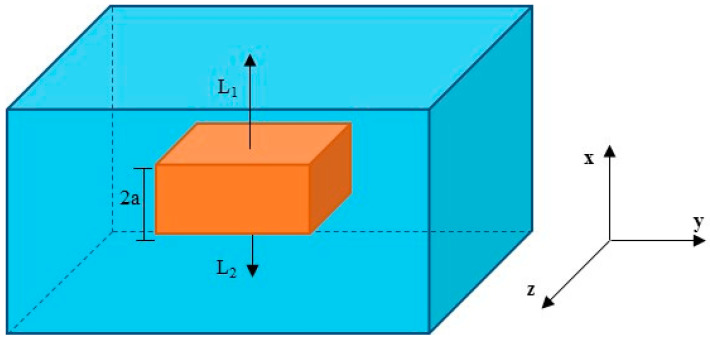
Geometric representation of the studied physical problem.

For the physical problem, the water absorption process was considered in a porous material (polymer composite) with a thickness of 2a, immersed in a fluid solute (water), at a specific temperature. The sample was located in such a way that the upper and bottom surfaces of the solid were at distances L_1_ and L_2_ from the upper and bottom walls of the water level in the container, as shown in Figure 1.

### 2.2. The Mathematical Model

For the analysis and solution of the mass transfer problem in the composite material, the following hypotheses were considered: (a) the material is considered homogeneous and isotropic; (b) the process is isothermal and transient; (c) there is no variation in the material dimensions; (d) the solid is considered completely dry at the beginning of the process; (e) the water transport inside the solid occurs only by one-dimensional diffusion; (f) there is an equilibrium condition between the external environment and the surface of the solid; (g) capillary transport through the solid is considered negligible; (h) there is no internal mass generation.

Langmuir’s model is an extension of the Fickian diffusion model. In this model, it is assumed that the migration of water inside the material occurs due to the existence of two phases—free and bound water molecules—which are represented mathematically by probability parameters. Based on these considerations, the Langmuir model is described by the following equations:

(1) $$\frac{\partial C}{\partial t}=\frac{\partial }{\partial x}\left(D\frac{\partial C}{\partial x}\right)+S^{C}$$

and

(2) $$S^{C}=\frac{\partial S}{\partial t}=\lambda C-\mu S$$

In Equation (1), C and S represent the free and bound water molecule concentrations inside the composite, respectively; D represents the free water molecule diffusivity (m^2^/s); S^C^ is the source term that describes the temporal variation of the bound water molecule concentration; λ is the probability that a free molecule will become bonded (s^−1^); μ is the probability that a bound water molecule will become free for diffusion (s^−1^); and t represents the time (s). For the solution of the governing equations, the following initial and boundary conditions were considered:

- Initial condition:

(3) $$S=C=0\;\left\{ \begin{array}{l} 0<x<2a\\ t=0 \end{array}\right.$$

- Boundary conditions:

(4) $$L_{1}\frac{\partial C}{\partial t}=-D\frac{\partial C}{\partial x}\;\left\{ \begin{array}{l} x=2a\\ t>0 \end{array}\right.$$

(5) $$L_{2}\frac{\partial C}{\partial t}=-D\frac{\partial C}{\partial x}\;\left\{ \begin{array}{l} x=2a\\ t>0 \end{array}\right.$$

According to the boundary conditions, it is assumed that the solute rate leaving the solution is equal to the solute diffusive flux at the surface of the composite. These conditions together allow us to evaluate the effect of the water level that surrounds the composite material on the water absorption kinetics.

The total moisture inside the material at a specific position x and in an instant of time t (local moisture) is given by the sum of the values of C and S at the same point, as follows:

(6) M=C+S

Once M is determined at any point within the composite, the average moisture content of the solid porous material at any time is given from the integration of Equation (6) in the volume of the solid. Since the problem is being investigated from a one-dimensional perspective, the equation to calculate the average moisture content assumes the following form:

(7) $$\bar{M}=\frac{1}{V}\int MdV=\frac{1}{2a}\int_{0}^{2a}Mdx$$

where 2a corresponds to the solid thickness.

### 2.3. The Numerical Solution

To obtain the numerical solution of Equations (1) and (2), the finite-volume method was used. In this method, the solution is obtained from the integration of the governing equations in the control volume of the domain and in time.

**Figure 2:**
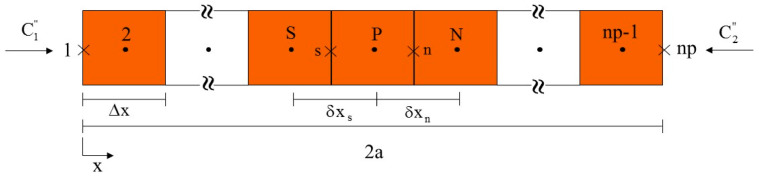
Schematic representation of the computational domain.

For the discretization process, a solid with uniform 2a thickness and (np-2) control volumes was considered, as illustrated in Figure 2. The solid was divided into three different regions: (a) internal control volumes; (b) upper boundary control volumes; (c) bottom boundary control volumes. Figure 2 shows nodal point P (in the center of the control volume), its south (S) and north (N) neighbors, the dimension of the control volume, the distances between nodal point P and its neighbors S ($\delta x_{s}$) and N ($\delta x_{n}$), and specific water fluxes on the bottom face $C_{1}^{\prime \prime }$ and top face $C_{2}^{\prime \prime }$ of the solid.

#### 2.3.1. The Free Molecule Concentration

- Internal points

Applying the integral in Equation (1) in the control volume of nodal point P over time, the following is obtained:

(8) $$\int_{x}\int_{t}\frac{\partial C}{\partial t}dtdx=\int_{t}\int_{x}\frac{\partial }{\partial x}\left(D\frac{\partial C}{\partial x}\right)dxdt-\int_{x}\int_{t}S^{C}dtdx$$

After the integration process, assuming a fully implicit formulation, Equation (8) takes the following form:

(9) $$\left(C_{P}-C_{P}^{o}\right)\frac{\Delta x}{\Delta t}=D_{n}\left(\frac{C_{N}-C_{P}}{\delta x_{n}}\right)-D_{s}\left(\frac{C_{P}-C_{S}}{\delta x_{s}}\right)-\left(\lambda C_{P}-\mu S_{P}^{o}\right)\Delta x$$

Rearranging the terms of Equation (9), the following discretized linear algebraic equation is obtained:

(10) $$A_{P}C_{P}=A_{N}C_{N}+A_{S}C_{S}+A_{P}^{o}C_{P}^{o}+SC$$

where the coefficients are given by:

(11) $$A_{P}=\left(\frac{\Delta x}{\Delta t}+\frac{D_{n}}{\delta x_{n}}+\frac{D_{s}}{\delta x_{s}}+\lambda \Delta x\right)$$

(12) $$A_{N}=\left(\frac{D_{n}}{\delta x_{n}}\right)$$

(13) $$A_{S}=\left(\frac{D_{s}}{\delta x_{s}}\right)$$

(14) $$A_{P}^{o}=\left(\frac{\Delta x}{\Delta t}\right)$$

(15) $$SC=\left(\mu \Delta x\right)S_{P}^{o}$$

Equation (10) is valid for all internal nodal points of the domain, except for boundary volumes (upper and bottom). The coefficients $A_{P}$, $A_{N}$, and $A_{S}$ represent the contributions of diffusive transport between point P and its corresponding neighbors. The term $A_{P}^{o}$ represents the influence of the C variable value in the previous time over its value at the current time.

- Control volumes for the upper boundary

For this control volume, there is a diffusive flux at the upper border, which must be replaced according to the considered boundary condition. In this type, the water flux at the upper border is described by Equation (4). Considering the diffusive flux at the control volume interface, and making the necessary substitutions, we obtain the following expression for the diffusive flux at the upper border:

(16) $$C_{1}^{\prime \prime }=\left(\frac{\frac{D_{n}}{\delta x_{n}}C_{P}-\frac{D_{n}}{\delta x_{n}}C_{n}^{o}}{1+\frac{D_{n}\Delta t}{\delta x_{n}L_{1}}}\right)$$

Thus, after the discretization process of Equation (1), the following type of control volume is obtained:

(17) $$\left(C_{P}-C_{P}^{o}\right)\frac{\Delta x}{\Delta t}=-C_{1}^{\prime \prime }-D_{s}\left(\frac{C_{P}-C_{S}}{\delta x_{s}}\right)-\left(\lambda C_{P}-\mu S_{P}^{o}\right)\Delta x$$

Substituting Equation (16) into Equation (17) and rearranging the terms, the following discretized linear algebraic equation is obtained (valid for the nodal point of the upper border):

(18) $$A_{P}C_{P}=A_{S}C_{S}+A_{n}^{o}C_{n}^{o}+A_{p}^{o}C_{p}^{o}+SC$$

where

(19) $$A_{P}=\left(\frac{\Delta x}{\Delta t}+\frac{1}{\frac{\delta x_{n}}{D_{n}}+\frac{\Delta t}{L_{1}}}+\frac{D_{s}}{\delta x_{s}}+\lambda \Delta x\right)$$

(20) $$A_{S}=\left(\frac{D_{s}}{\delta x_{s}}\right)$$

(21) $$A_{n}^{o}=\left(\frac{1}{\frac{\delta x_{n}}{D_{n}}+\frac{\Delta t}{L_{1}}}\right)$$

(22) $$A_{S}=\left(\frac{D_{s}}{\delta x_{s}}\right)$$

(23) $$SC=\left(\mu \Delta x\right)S_{P}^{o}$$

- Control volumes for the bottom boundary

The valid equation for this type of control volume is obtained in a similar way to Equation (18). However, for this case, the diffusive flux at the bottom boundary is considered, which must be replaced according to the boundary condition established in Equation (5). Thus, after all mathematical manipulations, the discretized Equation (5) takes the following form:

(24) $$C_{2}^{\prime \prime }=\left(\frac{\frac{D_{s}}{\delta x_{s}}C_{P}-\frac{D}{\delta x_{s}}C_{S}^{o}}{1+\frac{D_{s}\Delta t}{\delta x_{s}L_{2}}}\right)$$

Performing all the numerical procedures described previously, the discretized linear algebraic equation valid for the nodal point of the bottom boundary control volume will be given by:

(25) $$A_{P}C_{P}=A_{N}C_{N}+A_{S}^{o}C_{S}^{o}+A_{P}^{o}C_{P}^{o}+SC$$

where

(26) $$A_{P}=\left(\frac{\Delta x}{\Delta t}+\frac{1}{\frac{\delta x_{s}}{D_{s}}+\frac{\Delta t}{L_{2}}}+\frac{D_{n}}{\delta x_{n}}+\lambda \Delta x\right)$$

(27) $$A_{N}=\left(\frac{D_{n}}{\delta x_{n}}\right)$$

(28) $$A_{S}^{o}=\left(\frac{1}{\frac{\delta x_{s}}{D_{s}}+\frac{\Delta t}{L_{2}}}\right)$$

(29) $$A_{P}^{o}=\left(\frac{\Delta x}{\Delta t}\right)$$

(30) $$SC=\left(\mu \Delta x\right)S_{P}^{o}$$

Equations (10), (18), and (25), applied to their respective nodal points of the control volumes, form a system of equations whose solution indicates the free water molecule concentration within the composite material during the moisture absorption process.

#### 2.3.2. The Bonded Molecule Concentration

The discretized equations for the bonded solute concentration inside the solid is obtained using the analogous procedure for the free water molecule concentration. However, for this case, an explicit formulation was used to estimate the values. Thus, integrating Equation (2) into the volume and time period, the following expression is obtained:

(31) $$\left(S_{P}-S_{P}^{o}\right)\frac{\Delta x}{\Delta t}=\left(\lambda C_{P}-\mu S_{P}\right)\Delta x$$

Thus, reorganizing the terms of Equation (31), the following discretized linear algebraic equation is obtained:

(32) $$A_{P}S_{P}=A_{p}^{o}S_{P}^{o}+SS$$

where

(33) $$A_{P}=\left(\frac{\Delta x}{\Delta t}+\mu \Delta x\right)$$

(34) $$A_{P}^{o}=\left(\frac{\Delta x}{\Delta t}\right)$$

(35) $$SS=\lambda \Delta xC_{P}$$

Equation (32) is valid for all control volumes of the studied domain.

#### 2.3.3. Average and Local Moisture Contents

The local moisture content is obtained from the sum of the free and bound water molecule concentrations at each nodal point according to Equation (6). The average moisture content of the composite at each time point is obtained from the process of discretization of Equation (7), assuming the following form:

(36) $$\bar{M}=\frac{1}{2a}\sum_{i=2}^{np-1}M_{i}\Delta x_{i}$$

where np represents the number of nodal points used in the simulation. Thus, np-2 represents the number of control volumes in the studied domain.

#### 2.3.4. Numerical Simulation Procedure

To solve the discretized equations, a computational code was developed on the *Mathematica*^®^ platform. As the numerical results obtained from a given mathematical model were strongly dependent on the geometry, boundary conditions, the number of points in the mesh, and the time step considered, studies of the mesh refining and time step were performed. From this study, a numerical mesh with 30 nodal points (np = 30) and a time step of 20 s (Δt=20s) was chosen. The algebraic equation system obtained from Equation (25) was solved iteratively using the Gauss–Seidel method, where the following convergence criterion was assumed at each nodal point:

(37) $$\left|C_{P}^{n+1}-C_{P}^{n}\right|\leq 10^{-10}$$

where n represents the nth iteration for each time point.

#### 2.3.5. Evaluated Cases

In order to simulate the water absorption behavior inside the composite material and to evaluate the influence of the distance between the surface of the composite and the surface of the water layer in this process, different arbitrary cases were considered, as specified in Table 1. The absorption process was simulated considering an equilibrium moisture content of Me = 0.14488 kg/kg and a total process time of approximately 2250 h, equivalent to approximately 94 days. The values for the process parameters used in the simulations were defined based on the results reported in the literature [24,25]. 

## 3. Results and Discussions

In this research, the water absorption process in vegetable-fiber-reinforced polymer composites was studied. The focus was to analyze the effect of the distance between the tested sample surface and the water level in the container used; that is, the influence of the layer thickness water in the sorption process. Thus, the main idea is to offer a better interpretation of the experimental data, and consequently a more detailed description of the physical phenomenon.

**Figure 3:**
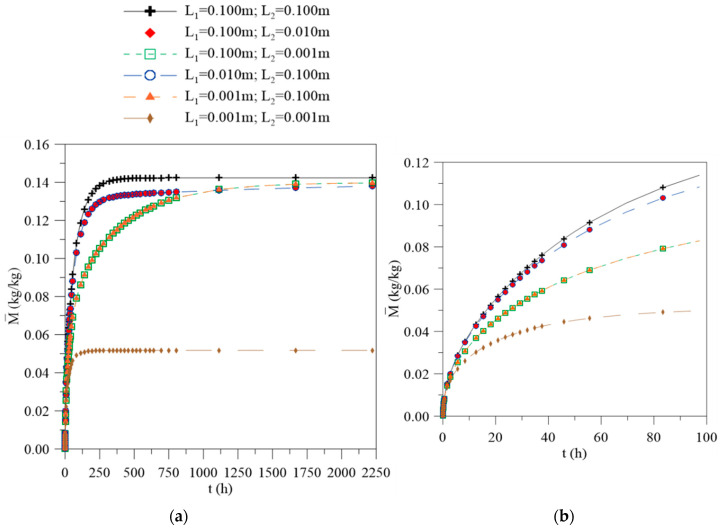
(**a**) Average moisture content as a function of the process time for different physical saturations. (**b**) Detailed view of (**a**).

Figure 3 illustrates the graph of the average moisture content in the material along the process for different arbitrary cases, as reported in Table 1. During the absorption process, water tends to penetrate into the material through the concentration gradient until reaching the saturation condition, whereby the equilibrium with the external environment is reached. From analysis of Figure 3, it is possible to observe that the water absorption is faster in the initial process, increasing almost linearly until a state known as “pseudo equilibrium”, which is characteristic of the absorption behavior predicted by the Fickian diffusion law. Following this, the absorption curve begins to assume a concave shape in relation to the abscissa axis. With continuous exposure of the composite to the water, the process becomes slower until reaching the hygroscopic saturation point, which varies according to the previously established physical and geometric conditions. It is possible to verify that the water layer thickness close to the composite surface strongly affects the water absorption process. This effect is not restricted only to the water migration rate into the material, but also the maximum amount absorbed by it.

For the case 1 (L_1_ = 0.1 m e L_2_ = 0.1 m), the average moisture content tends towards the equilibrium moisture content (0.1423 kg/kg), whereas for the case 6 (L_1_ = 0.001 m e L_2_ = 0.001 m), the value approaches 0.0517 kg/kg. This behavior happens due to the difference in the water amount available for the diffusive process in contact with the composite surface; that is, the change in the composite water level distance causes variations in the absorption kinetics that can be explained for the concentration gradient behavior on the composite surface. This phenomenon can be understood when separately plotting the graphs for the free water molecule concentration, bound water molecule concentration, and local moisture as a function of the position inside the material.

**Figure 4:**
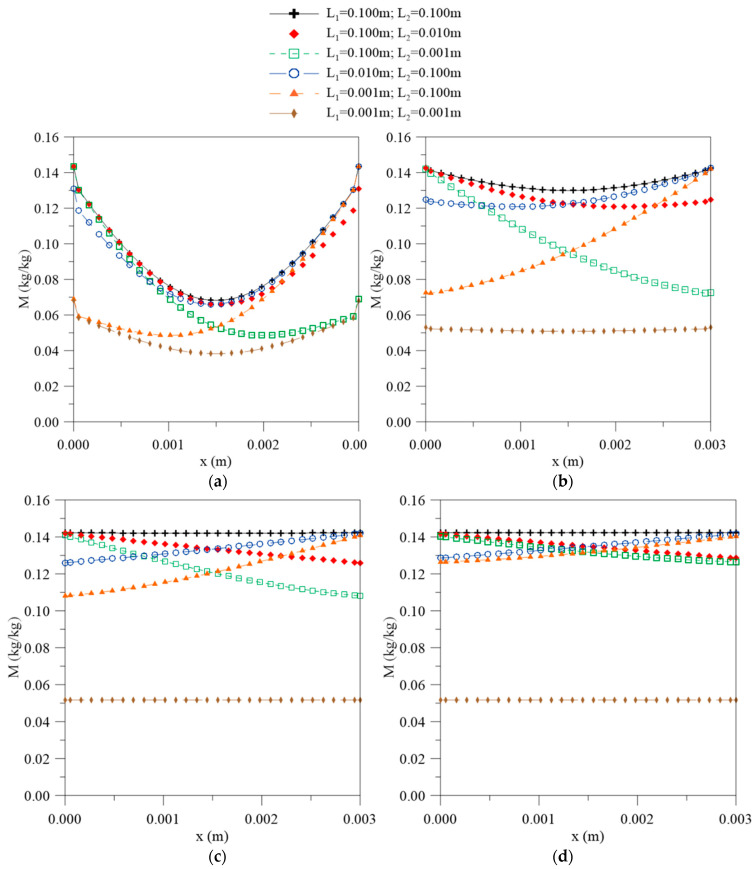
Local moisture content as function of the position inside the material at different process times: (**a**) 56, (**b**) 195, (**c**) 500, and (**d**) 1944 h.

Figure 4 shows the local moisture contents obtained from the sum of the free and bound water molecule concentration along the thickness of the material for different process times and different simulated cases. From the analysis of this figure, it is possible to observe that the largest moisture content gradients are found on the surfaces. In those regions the absorption is faster due to the higher concentration of water, which then decreases towards the central region of the solid, showing increased immersion time until reaching a characteristic equilibrium state for each simulated case. Symmetry can also be observed in the results when the geometric parameters L_1_ and L_2_ are equal.

**Figure 5:**
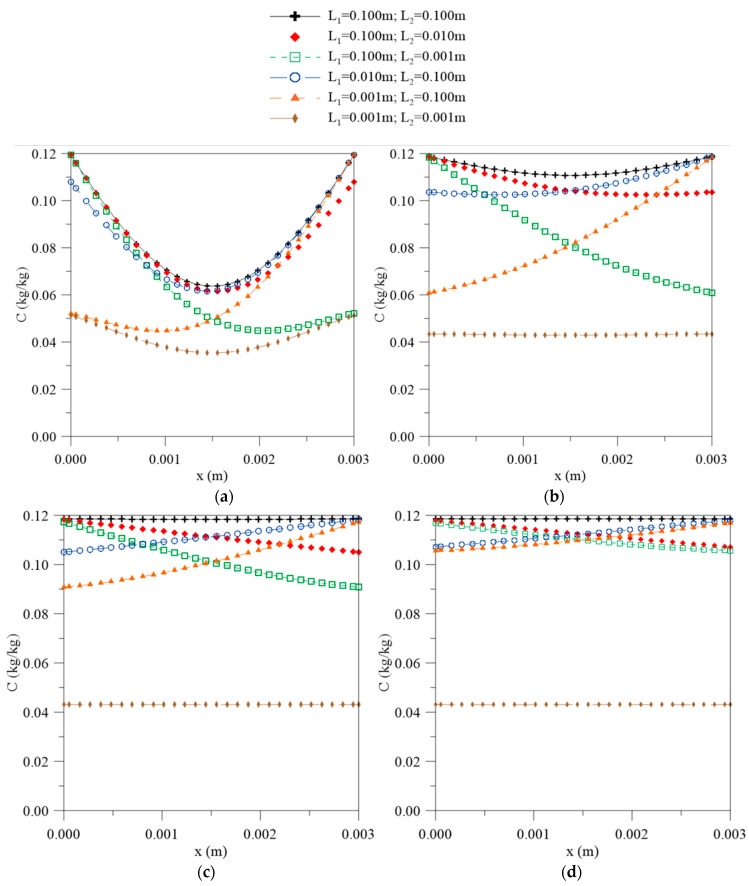
Free water molecule concentration as a function of the position inside the material at different process times: (**a**) 56, (**b**) 195, (**c**) 500, and (**d**) 1944 h.

**Figure 6:**
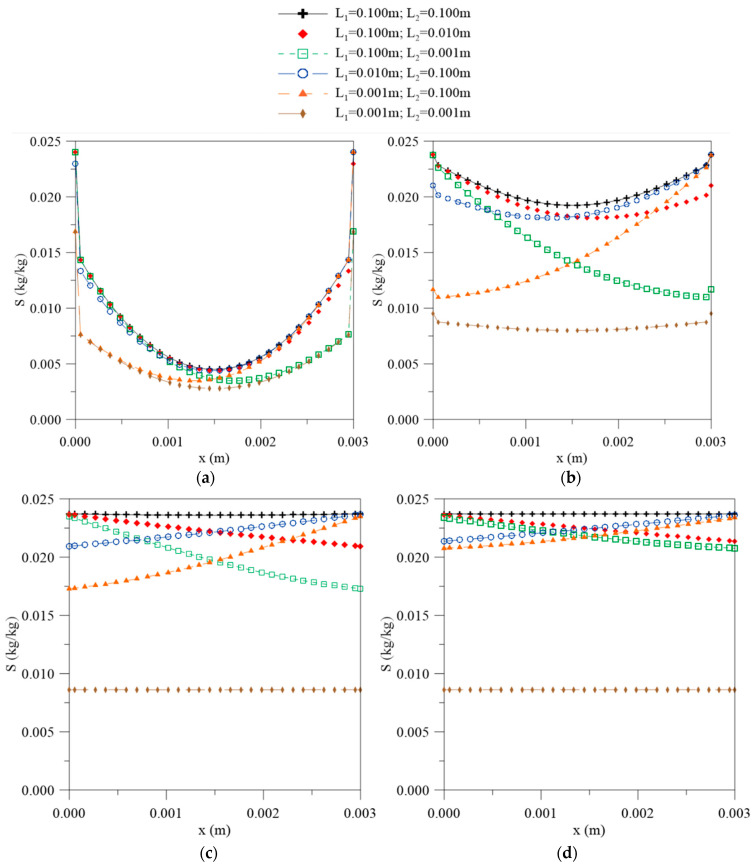
Bound water molecule concentration as a function of the position inside the material at different process times: (**a**) 56, (**b**) 195, (**c**) 500, and (**d**) 1944 h.

Figure 5 and Figure 6 illustrate the transient behavior of free and bound water molecules in the material along the thickness for different process times. In Figure 5, it can be observed that increasing the immersion time causes changes in the diffusion behavior of the free water molecules. For the initial times (Figure 5a), the curve follows symmetrical behavior, with the highest concentration gradients at the surface of the material. With the increase in exposure time, the curve assumes an asymmetric behavior that depends on the previously specified values (Figure 5b–d), causing variations in the concentration gradient.

The bound water molecule concentration along the material, in turn, is dependent on the process time, which varies according to the free molecule concentration. From the analysis of Figure 6, it can be observed that the largest concentration gradients of the bound water molecules are present on the material surfaces, mainly for cases 1 and 6, due to the symmetry of the process. For other cases, the curve behavior changes according to the water layer thickness on the bottom and upper surfaces of the composite material. In all cases, the process occurs until reaching equilibrium. The time taken is dependent on the physical and geometric conditions established. It is important to note that Langmuir’s model predicts that the saturation balance is attained when the following condition is satisfied:

(38) λC=μS

The condition (Equation (38)) was proven in this study for all cases analyzed. The hygroscopic saturation for cases 1 and 6 were achieved in 1944 and 500 h, respectively. In these times, the concentrations of free water (Ce) and bound water (Se) molecules in the equilibrium condition were 0.1185 and 0.0238 for case 1, and 0.0431 and 0.0086 for case 6, respectively. Another important result is related to the absolute values of the free and bound water molecule concentrations. Analyzing Figure 5 and Figure 6, it can be seen that C >> S was ensured at all points of the domain and any process times, especially in the final stages of process.

Different studies agree that the water diffusion in polymer composites occurs via three different mechanisms. First, water molecules diffuse through defects present in the matrix, such as microcracks and pores. Then, the diffusion continues by capillarity along the interface, infiltrating in the different hydrophilic components of the fibers and finally penetrating the hollow fiber parts, causing swelling. This effect results in the break of hydrogen bonds between molecules in the fiber; thus, the hydroxyl groups or other polar groups form new bonds with water molecules. This phenomenon causes an increase in the free volume of the structure, and consequently a reduction in the properties of the materials produced [6,22,26].

The concentration gradients of the free and bound molecules and local moisture have huge influences on the diffusive process behavior; some values for these parameters are reported in Table 2. The concentration gradient represents the water molecules flux across a frontier; therefore, the variation in this parameter depends on the contact area between the water and the composite material. From the analysis of Table 2, it is observed that the higher gradients are found in the regions where L assumes a higher value; thus, for the cases where L_1_ >> L_2_, the diffusion will occur predominantly in the direction of L_1_ and vice versa. For the cases where the distances between L_1_ and L_2_ are equal, there is no change in its values; that is, the concentration variation rate is the same on both surfaces and the symmetry of the phenomenon is verified. With the increase in the process time, the values of these parameters decrease, as observed in Figure 4, Figure 5 and Figure 6. It is interesting to state that according to Figure 3, no changes in the absorption curve were verified in the reciprocal cases (cases 2–5). This fact can be better understood from the analysis of the gradient behavior. For these cases, only a change in the moisture flux direction occurs due to the orientation of the x axis; modification of the average moisture content inside the solid was not verified, rather the moisture content distribution and the free and bound water concentrations distributions were modified. 

Furthermore, according to Table 2, the concentration gradients of the bound water molecules are higher than the free water molecules gradients in most of the process, mainly for short times.

**Figure 7:**
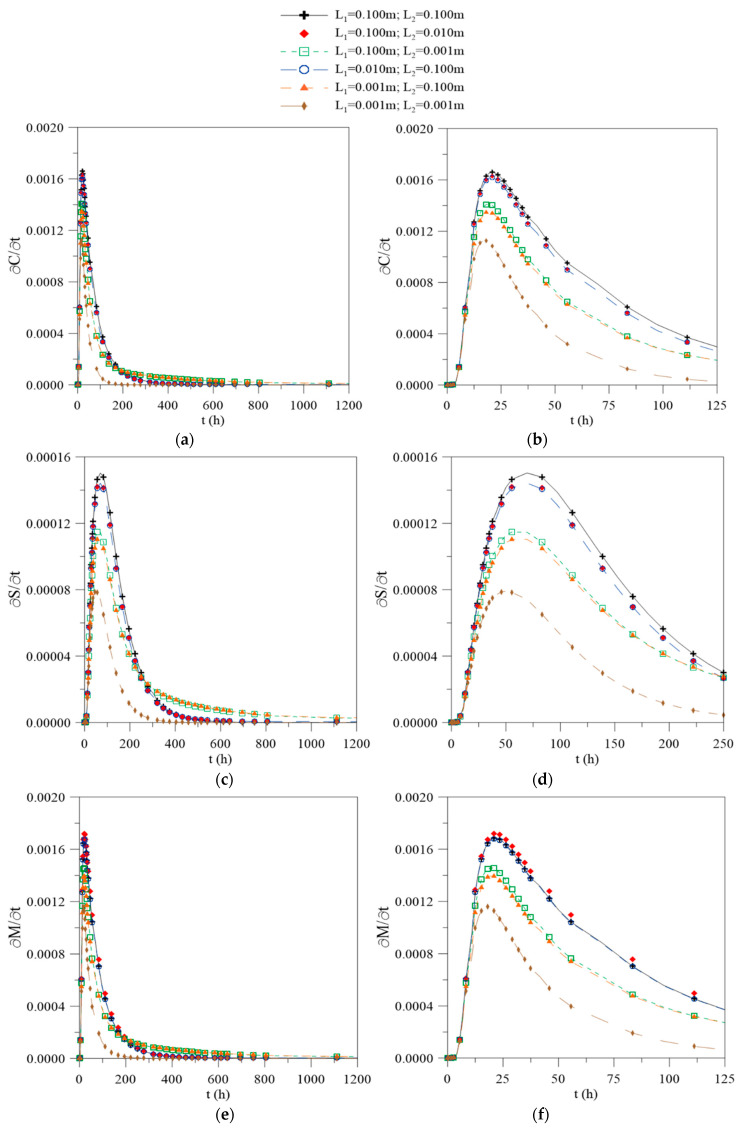
Time variation rate of the (**a**) free water molecules and (**b**) detailed view of (**a**). (**c**) Bound water molecule concentration and (**d**) detailed view of (**b**). (**e**) Local moisture content in the center of the composite and (**f**) detailed view of (**e**).

Figure 7 shows the behavior of the variation rates of free (Figure 7a) and bound (Figure 7b) water molecules and the local moisture content (Figure 7c) as a function of time in the center of the composite. From the analysis of the figures, it is possible to observe that the water absorption process is faster at the beginning of the process until reaching a maximum absorption point, followed by a decrease that tends towards the saturation equilibrium point. 

In the initial time period, the material starts to moisten at an increasing rate, because it is completely dry at the beginning of the process. After reaching an absorption peak, these values start to drop, the material starts to soak, and the water starts to diffuse with more difficulty, slowing down the process.

For case 1, it is observed that the material presents a maximum absorption point at around 22 h, where it accumulates a local moisture percentage of about 40%. The free water concentration reaches a peak at around 21 h, while that bound water molecules reach a peak at around 70 h; thus, in comparison, the bound water molecules have a lower variation rate than the free water molecules. However, in the equilibrium state, the point where the material reaches its absorption capacity is only reached after 2250 h. This behavior complements the theories reported in the literature. According to Chenetal [27], in the absorption process, the free water has the ability to move independently through the empty spaces, while the bound water is restricted to polar groups of the material, which explains the difference in the maximum times obtained.

For case 6, the maximum peak was observed at around 18 h, with 66% of the local moisture content percentage being achieved due to the lower amount of water available for diffusion. For the other cases, there is a smaller variation in the maximum absorption times. However, there is a difference in the moisture content values obtained of about 18% between the reciprocal cases (cases 2–5), reinforcing the idea that the established process conditions and the experimental variations, in terms of the sample positions in gravimetric tests, influenced the water absorption process behavior.

In order to verify the composite water absorption behavior, from the results provided by the Langmuir’s model, the temporal variation rates of C, S, and M for the initial and maximum values were obtained and are reported in Table 3. From the analysis of this table, it was concluded that the maximum temporal variation rate of the free water molecule concentration was approximately 10 times greater than for bound water molecules. Another important point is that the maximum variation rate ${\partial C/\partial t|}_{\max}$ occurs on the first day of the process, whereas ${\partial S/\partial t|}_{\max}$ occurs with a certain delay on the third day of the process. Furthermore, it was found that the maximum C value is approximately 87.3% of the local moisture content M, which explains the fact that in many situations, the Fick’s model adequately predicts the process from a mathematical point of view.

## 4. Conclusions

This research presented an advanced one-dimensional, isothermal, and transient mathematical model that predicts the water absorption process in vegetable-fiber-reinforced polymer composites (Langmuir-type model). The proposed model complements previous work, because it considers the effect of the water layer thickness on the material surface. 

From the numerical results obtained using the finite volume method, it can be concluded that:

(a) The water absorption is faster at the initial time points of the process, and with continuous exposure the process becomes slower until reaching the hygroscopic saturation point, which varies according to the physical and geometrical parameters;
(b) The water layer thickness on the composite surface strongly affects the moisture absorption kinetics and the moisture content gradients inside the material. The higher gradients are found in the regions where L_1_ or L_2_ has a higher value.
(c) The largest concentration gradients of free and bound water molecules are found on the composite surface, and a symmetric behavior is found when the geometric parameters L_1_ and L_2_ are equal;
(d) The equilibrium moisture content of the composite is dependent on the water layer thickness on the material surface. For L_1_ = L_2_ = 0.100 m, the average moisture content tends towards moisture equilibrium content of approximately 0.1423 kg/kg, whereas for L_1_ = L_2_ = 0.001 m this value approaches 0.0517 kg/kg, due the small quantity of water available for diffusion;
(e) The water absorption process is faster at the beginning of the process, until reaching a maximum absorption point, after which it decreasing until reaching saturation equilibrium. For L_1_ = L_2_ = 0.100 m, the material presents a maximum absorption point at around 22 h, where it accumulates a local moisture percentage of about 40%, meanwhile for L_1_ = L_2_ = 0.001 m the maximum peak is observed around 18 h of process, with 66% local moisture;
(f) The values of the free water molecules are higher than for bound water molecules at all points of the domain and at any process times, especially in the final stages of the process;
(g) The temporal variation rate of the free water molecule concentration is higher than the bound water molecule concentration in the center of the sample. The maximum variation rate for free water molecules occurs on the first day of the process and for the bound water molecules occurs after two days.

Thus, this research strongly contributes to the field of composite materials, providing detailed information from mathematical and physical perspectives on the water absorption behavior, and consequently its effects on the product lifetime during operation. Therefore, this information will assist engineers and researchers to more efficiently design products based on vegetable fibers for specific applications.

## Figures and Tables

**Table 1:** Process parameters (Temperature, thickness, probabilities and diffusivity) values used in the simulations.

Case	Parameter							
	Water	Composite						
	Te (°C)	To (°C)	2a (m)	L_1_ (m)	L_2_ (m)	μ (10^−6^s^−1^)	λ (10^−6^s^−1^)	D (10^−12^m^2^s^−1^)
1	25	25	0.003	0.100	0.100	5	1	5
2	25	25	0.003	0.100	0.010	5	1	5
3	25	25	0.003	0.100	0.001	5	1	5
4	25	25	0.003	0.010	0.100	5	1	5
5	25	25	0.003	0.001	0.100	5	1	5
6	25	25	0.003	0.001	0.001	5	1	5

Te: Water bath temperature. To: Initial temperature.

**Table 2:** Values for the concentration gradients of the free and bound water molecules and the local moisture content at the surface of the material for each simulated case.

t (h)		x (m)	Case					
			L_1_ = 0.100m L_2_ = 0.100m	L_1_ = 0.100m L_2_ = 0.010m	L_1_ = 0.100m L_2_ = 0.001m	L_1_ = 0.010m L_2_ = 0.100m	L_1_ = 0.001m L_2_ = 0.100m	L_1_ = 0.001m L_2_ = 0.001m
56	∂C/∂x	0.000	−61.30	−61.94	−66.07	−51.11	−9.79	−12.88
		0.003	61.30	51.11	9.790	61.94	66.10	12.88
	∂S/∂x	0.000	−180.53	−180.55	−180.7	−179.2	−172.26	−172.40
		0.003	180.53	179.20	172.3	180.5	180.73	172.40
	∂M/∂x	0.000	−241.83	−242.50	−246.8	−230.3	−182.10	−185.28
		0.003	241.83	230.30	182.1	242.5	246.81	−185.28
194	∂C/∂x	0.000	−8.610	−12.56	−27.51	−2.289	7.026	−0.4140
		0.003	8.610	2.290	−7.060	12.56	25.51	0.4140
	∂S/∂x	0.000	−18.02	−18.61	−21.11	−16.56	−13.11	−14.450
		0.003	18.02	16.56	13.11	18.606	21.11	14.450
	∂M/∂x	0.000	−26.63	−31.17	−48.61	−18.85	−6.082	−14.860
		0.003	26.63	18.85	6.090	31.17	48.61	14.860
500	∂C/∂x	0.000	−0.190	−4.730	−11.57	3.811	3.702	−0.0020
		0.003	0.190	−3.810	−3.702	4.733	11.56	0.0020
	∂S/∂x	0.000	−0.180	−1.010	−2.773	0.670	0.799	−0.0610
		0.003	0.180	−0.670	−0.799	1.097	2.773	0.0610
	∂M/∂x	0.000	−0.370	−5.830	−14.34	4.481	4.501	−0.0620
		0.003	0.370	−4.481	−4.501	5.830	14.34	0.0620
1944	∂C/∂x	0.000	−0.003	−3.769	−4.867	3.226	1.564	−5.41 × 10^−6^
		0.003	0.003	−3.226	−1.564	3.769	4.867	5.41 × 10^−6^
	∂S/∂x	0.000	−0.002	−0.781	−1.137	0.666	0.365	−0.00015
		0.003	0.002	0.666	−0.365	0.781	1.137	0.00015
	∂M/∂x	0.000	−0.005	−4.551	−6.004	3.892	1.929	−0.00016
		0.003	0.005	−3.892	−1.929	4.551	6.004	0.00016

**Table 3:** Values for the concentrations of C, S, and M, and the maximum absorption value at the center of the composite.

Case	t (h)	C (kg/kg)	∂C/∂t (kg/kg/s)	t (h)	S (kg/kg)	∂S/∂t (kg/kg/s)	t (h)	M (kg/kg)	∂M/∂t (kg/kg/s)
1	20.8	0.01918	0.00166	69.4	0.00659	0.00015	22.2	0.02197	0.00172
2	20.8	0.01892	0.00163	69.4	0.00641	0.00014	20.8	0.01932	0.00169
3	18.1	0.01310	0.00141	55.6	0.00372	0.00011	19.4	0.01535	0.00146
4	19.4	0.01659	0.00162	69.4	0.00638	0.00014	20.8	0.01923	0.00168
5	18.1	0.01252	0.00135	55.6	0.00357	0.00011	19.4	0.01467	0.00140
6	16.7	0.00948	0.00113	45.8	0.00202	7.9 × 10^−5^	18.1	0.01124	0.00116
